# Ambulance staff awareness of vitamin D and risk of deficiency in a UK ambulance service: a survey-based evaluation

**DOI:** 10.29045/14784726.2021.3.5.4.65

**Published:** 2021-03-01

**Authors:** Larissa Prothero

**Affiliations:** East of England Ambulance Service NHS Trust

## Abstract

**Aims:**

The aim of this service evaluation was to explore staff awareness of vitamin D and the risks associated with deficiency in the ambulance setting, to inform the need for appropriate well-being support and resources.

**Methods:**

An online, anonymous 20-question survey was purpose-designed, based on a validated vitamin D questionnaire. It was made available to staff for completion in one UK ambulance service between 16 June and 12 July 2020, resulting in a convenience sample of 354 responses. These were analysed using quantitative (descriptive) and qualitative (thematic) approaches.

**Results:**

The cohort age range was 20 to 65+ years; 41% (n = 156) were male. Over half of respondents worked within emergency operational service delivery (57%; n = 219), but all key service roles were represented. Respondents reported to be predominantly ‘White British’ (92%; n = 352), but nine ethnic groups were included. According to the Fitzpatrick Scale, most staff described themselves as having a ‘Medium, between white to moderate brown: sometimes mild burns, gradual tan’ complexion (47%; n = 182) or ‘White, Fair: usually burns, tans with difficulty’ (32%; n = 124). The majority felt they got sufficient sunlight exposure when at home (66%; n = 253), but not at work (58%; n = 222). Typically, respondents stated they were ‘neither unconcerned or concerned’ about having vitamin D deficiency (35%; n = 135), although almost a fifth (17%; n = 66) reported to have been told they had vitamin D deficiency by a medical professional. Forty percent of respondents took vitamin D supplements: 12% (n = 45) as advised by a medical professional, and 28% (n = 107) self-directed to prevent vitamin D deficiency. Of the remaining, many (35%; n = 136) had not considered taking such dietary supplements. The ability of respondents to recognise known factors that affect vitamin D production in the skin, good vitamin D food sources and individuals at risk of vitamin D deficiency was variable. Additional comments raised by respondents related to lack of vitamin D awareness, the risk factors and impact of deficiency, vitamin supplementation, COVID-19, work arrangements and sunlight exposure, self-learning and general well-being.

**Conclusion:**

There is scope for improved awareness of vitamin D and the risks for deficiency in the ambulance setting. It appears service staff are at risk of vitamin D deficiency irrespective of their role: access to sunlight and use of vitamin D supplements are variable. The physical and mental impacts of deficiency can be significant, requiring absence from work. The development of appropriate vitamin D and well-being resources appears to be warranted.

